# Identifying Markers of Dignity-Conserving Care in Long-Term Care: A Modified Delphi Study

**DOI:** 10.1371/journal.pone.0156816

**Published:** 2016-06-15

**Authors:** Genevieve N. Thompson, Jennifer McArthur, Malcolm Doupe

**Affiliations:** 1 College of Nursing, Rady Faculty of Health Sciences, University of Manitoba, Winnipeg, MB, Canada; 2 Manitoba Palliative Care Research Unit, CancerCare Manitoba, Winnipeg, MB, Canada; 3 Faculty of Nursing, University of Alberta, Edmonton, AB, Canada; 4 College of Medicine, Rady Faculty of Health Sciences, University of Manitoba, Winnipeg, MB, Canada; University of Exeter, UNITED KINGDOM

## Abstract

Ensuring that people living in nursing homes (NHs) are afforded with dignity in their daily lives is an essential and humane concern. Promoting dignity-conserving care is fundamentally important. By nature, however, this care is all-encompassing and holistic, and from current knowledge it is challenging to create explicit strategies for measuring dignity-conserving care. In practice the majority of current NH indicators of quality care are derived from information that is routinely collected on NH residents using the RAI-Minimum Data Set (MDS). In this regard, issues that are more tangible to resident dignity such as being treated with respect, compassion, and having opportunities to engage with others are not adequately captured in current NH quality of care indicators. An initial set of markers was created by conducting an integrative literature review of existing markers and indicators of dignity in the NH setting. A modified Delphi process was used to prioritize essential dignity-conserving care markers for use by NH providers, based on factors such as the importance to fostering a culture of dignity, the impact it may have on the residents, and how achievable it is in practice. Through this consensus building technique, we were able to develop a comprehensive set of markers that capture the range and diversity of important dignity-conserving care strategies for use in NHs. The final 10 markers were judged as having high face validity by experts in the field and have explicit implications for enhancing the provision of daily dignified care to NH residents. These markers make an important addition to the traditional quality indicators used in the NH setting and as such, bridge an important gap in addressing the psychosocial and the less easily quantified needs of NH residents.

## Introduction

Ensuring that people living in nursing homes (NHs) are afforded with respect and dignity in their daily lives is an essential and humane concern. However, providing dignity-conserving care can be challenging within this milieu owing to constraints on care providers’ time, resources, and the proclivity towards task driven care. Additionally, the concept of dignity-conserving care is complex and subjective [1–3], leading to differences in opinions as to what constitutes dignified care. For example, care providers may feel that they provide dignified care if they undertake certain care tasks for the resident, while residents may focus more on the manner in which this care was delivered [4]. The research literature describes many instances where, though care is provided in a technically proficient and competent manner, residents felt their dignity was assaulted since health care providers failed to provide compassionate, person-centered care [5–8]. Developing markers of dignity-conserving care has value for measuring the prevalence of and factors associated with providing this care in NH environments, for educating NH staff on the appropriate approaches to providing dignified care, and for ultimately improving the quality of life for NH residents.

Dignity is a common term used in everyday discourse. Some people argue that preserving resident dignity is one of the most important ethical considerations when providing care to a NH resident [9–11]. Dignity includes notions of being able to maintain feelings of physical comfort, autonomy, meaning, interpersonal connectedness, hopefulness, and belonging [12–15]. Dignity can also be influenced through the evaluation of our social interactions [16–18]. The notion that one’s sense of dignity is influenced either positively or negatively by others has been reported previously [19]. In essence, therefore, supporting resident dignity is much more than adhering to technical standards and clinical guidelines; at its core, dignity-conserving care is reflected in the process of providing care that meets the unique needs of the individual and is highly influenced by NH staff attitudes and behaviours [8].

Just as scientists have used consensus-building techniques to prioritize clinical markers of quality care and strategies to implement change in practice [20–23], in this paper we show how consensus building techniques can be used to identify markers of dignity-conserving care, that attend to both processes of care and the personal aspects of care which contribute to maintaining dignity. While there has been work, predominately in the United Kingdom, on developing markers of dignity in care, they are described as a high-level framework cutting across multiple sites of care [4]. While this type of framework is important, the purpose of this study was to develop a concise set of markers, fundamental to achieving dignity-conserving care within the NH environment.

## Methods

A modified Delphi process was used to prioritize essential dignity-conserving care markers, based on factors such as the importance to fostering a culture of dignity, the impact it may have on the residents, and how achievable it is in practice. The Delphi technique is a common method used in selecting healthcare quality indicators, as it allows a large number and variety of experts from across diverse geographic locations to be involved [24,25]. This structured process is conducted iteratively, giving participants the opportunity to compare, contrast, and modify their expert opinion, based on that provided by peers. Typically this iterative process continues until consensus is reached, for a maximum of three Delphi rounds [26]. Consistent with the strategies used by others [27], our process was modified from the traditional Delphi technique, which starts by soliciting open-ended questions to generate ideas around the topic of interest (i.e., asking participants to generate an initial list of dignity-conserving care markers. Like others however, during each Delphi round we encouraged participants to provide suggestions of additional dignity conserving care markers to be considered in our analysis. Also, following the procedures outlined by Keeney and colleagues [28], we conducted 3 Delphi rounds, such that participants received individualized feedback following the first and second rounds.

This study received ethical approval from the University of Manitoba Education/Nursing Research Ethics Board (ENREB), and from the Regional Health Authorities (RHAs) in which participants were recruited, including the Boards from Northern RHA, Prairie Mountain RHA, Interlake-Eastern RHA, and Southern Health. Written informed consent was obtained from all participants. This project was funded by the Government of Manitoba.

### Advisory Team

An advisory team was created for consultation purposes. This group did not participate in the actual Delphi rounds, but instead was consulted to assist in the recruitment of experts to participate in the Delphi process, and to review both the initial and final list of dignity-conserving care markers. This team (n = 12) included long-term care experts and policy-makers from the health branch of the provincial government (Manitoba Health), and at least one representative, who was responsible for the programs and policies in long-term care, from each of the 5 Provincial Regional Health Authorities. For a further description of the organization of long-term care in Manitoba, please see Doupe et al [29].

### Delphi Panellists

The formation of an “expert panel” is foundational to the Delphi method [28]. While representativeness is not expected as part of the Delphi process [25], the Advisory Team was invited to use their networks to purposefully select panellists who were key members within NHs, who were both direct and indirect care providers and represented the urban, rural, and remote geographic diversity of NHs in our province. An attempt was made to recruit participants in a proportional manner according to the number of NHs in each region. In this regard, members from the two largest health authorities nominated up to 20 individuals each and the remaining regions nominated up to 10 each. In total, 51 participants from a variety of facilities across the regions agreed to participate. Prior to data collection, participants were asked to complete a consent form, and a participant information form, which requested basic demographic information, including length of employment in long-term care, professional designation, gender, and current employment status.

### Framing an Initial List of Dignity Markers

An initial set of markers was created by conducting an integrative literature review of existing markers and indicators of dignity in the NH setting. This review was conducted independently and in parallel by multiple investigators such as a research librarian, a study Research Assistant, and a member of the research team (GT). Search engines for this phase of the research included MEDLINE, Scopus, CIHAL, Ageline, and PsychInfo databases using the following subject headings/key words: dignity, human dignity, indignity, nursing homes, nursing home patients, long term care, homes for the elderly. Identified articles were then categorized into four groups: 1) theoretical perspectives describing dignity and existing markers of dignity, 2) dignity according to NH residents and family of residents, 3) dignity according to nurses and NH staff, and 4) person-centeredness. One report played an especially important role in the creation of the initial list of markers, entitled “Measuring Dignity in Care for Older People: A Research Report for Help the Aged” by the Picker Institute (i.e. “The Picker report”) [4], which outlined a large number of dignity indicators for older people across settings of care, including the NH setting. Research articles that described elements of dignity supporting care specific to the NH environment bolstered the initial list. Additional articles were also found by reviewing articles found in the reference section of these initial articles.

A total of 63 potential markers were identified through this process. Based on the prior unpublished pilot work by GT examining dignity in NH care, the markers were grouped into four broad categories that were deemed representative of NH residents’ experiences of dignity: Being Known, Care and Assistance, Privacy, and Social Interaction. Briefly, the pilot work was conducted in 15 long-term care facilities in which separate round-table sessions (n = 22) were facilitated by GT with cognitively-well residents, family caregivers, and staff. The objective was to understand what supported or undermined a resident’s sense of dignity, what were the biggest challenges to supporting resident dignity, and what was perceived as being done right to support dignity. The detailed notes and audio-recordings of each session were thematically analyzed by two members of the research team to develop the four broad categories. “Being Known” represents activities centred around upholding the personhood of residents and accounting for personal preferences; “Care and Assistance” focuses on providing intimate personal care in a caring and compassionate manner; “Privacy” acknowledges the importance of respecting residents personal and physical space; and “Social Interaction” underscores the need for residents to have meaningful interactions both inside the facility and connect with the world beyond.

Once the initial list of (N = 63) markers was created, the Advisory Team reviewed the items for accuracy, clarity, and to provide feedback on the content and wording of the markers along with the accurate categorization of the markers. No markers were removed from the list or moved from the assigned category based on their responses; however the wording of some markers was altered.

#### Conducting the Delphi Process

In Round One of the Delphi process, participants were emailed a questionnaire containing the initial list of 63 dignity markers, sectioned into the four categories of Being Known (17 markers), Care and Assistance (15 markers), Privacy (17 markers), and Social Interaction (14 markers). Instructions were provided asking participants to answer questions as generally as possible, without thinking specifically about the facility where they worked. Markers were all worded so that they completed the sentence, “Dignity exists when…” (e.g., “dignity exists when residents have a choice of whether or not to attend activities”).

Participants were asked to rate each marker on its importance, achievability, and impact on resident dignity. Importance was assessed using the question, “How important is this to fostering a culture of dignity?” A Likert scale was used to rate this, where 1 = Not at all important, 2 = Not too important, 3 = Undecided, 4 = Somewhat important, and 5 = Extremely important. Achievability was measured using the question, “Is this achievable?” where 1 = Not at all, 2 = Yes, easily, and 3 = Yes, but not easily. Last, impact was assessed with the question, “What impact does this have on the resident?” A Likert scale was again used, where 1 = No impact, 2 = Minimal impact, 3 = Neutral, 4 = Moderate impact, and 5 = Large impact. An optional “Comments” section was also available for each marker, to allow participants to justify their decision, to clarify their understanding, or to make suggestions. All forms were returned to the researchers by email or fax.

During Round Two, participants were provided with the aggregate Round One scores for each dignity marker in comparison to their personal scores. As we found little to no variation in how participants scored dignity markers by their “impact” and “importance” in Round 1 (i.e., all were deemed to be highly important and impactful) during Round 2 participants were asked to re-score the remaining markers based on the principle of achievability only. In addition, participants were asked to identify their top 15 markers (i.e. markers they thought best represented dignity-conserving care in the NH setting). For informational purposes, participants were provided with the list of discarded markers, and the reasoning behind why they had been discarded. As in Round 1, an optional comments section was provided after each marker.

A third and final Delphi round was conducted to provide participants with the final list of markers and to give them the opportunity to comment on the list. Participants were notified that in Round 1, these markers rated high in importance, impact, and achievability, and in Round 2, at least 55% of participants included these markers in their top 15 selection. Participants were asked if the selected markers were representative of dignified care in the NH setting and to explain why. They were also instructed to indicate if any marker was missed that they thought should be included in the final list.

#### Data Analysis

In the first Delphi round, the average scores for importance, achievability, and impact, were reviewed to create cut-off points. These cut-offs were used to define markers that were rated as being less important, as having a lower impact on residents, or that were viewed as not being achievable to address by Delphi participants. Since most markers were generally rated very highly by participants, markers that achieved an overall average score of less than 4.70 for both importance and impact were discarded. Alternatively, markers were discarded if less than 40% of participants indicated that the marker was easily achievable. In Round Two, the average scores for achievability and the number of participants endorsing a marker as being in their top 15 were calculated. Responses provided by participants in the Third Delphi Round were qualitative in nature. All responses were read through by two members of the research team and coded for consensus on the dignity-conserving care markers. A list of markers that were identified by participants as ‘missing’ was collected and categorized.

## Results

A total of 63 dignity markers were selected from the literature and included in a preliminary set of dignity-conserving care markers. Of the 51 individuals who agreed to participate, 42 individuals completed Round 1, 37 participated in Round 2 and 36 in Round 3. Sixty-eight percent of panellist participated in all 3 rounds. A reminder email was sent to non-responders after each round encouraging them to participate. Of those who completed the demographic information (n = 41), 92% were female, respondents identified their educational background as Registered Nurses (n = 15), Social Workers (n = 6), Registered Psychiatric Nurses (n = 4), Dietician (n = 2) and Rehabilitation Therapists (n = 2). 88% were employed full-time, and had a mean length of employment in long-term care of 13.2 years.

In Round 1, using the cut-offs, 25 markers were discarded, and 38 markers were kept for further discussion (Table 1). Scores for these latter markers were summarized, and participant comments from these markers were reviewed for common themes and summarized, in preparation for our second Delphi round.

**Table 1:** Scoring of Round One Markers.

	Importance Average	Impact Average	Achievability, % No not at all (1)	Achievability % "Yes, easily" (2)	Achievability % Not easily achievable (3)	Discard if Importance and Impact rated < 4.70 OR > 60% Rated as not easily Achievable
**Being Known n = 17**						
Staff does not use elder-speak (e.g. using terms such as ‘dear’ or ‘sweetie’) [4, 7, 31,32,34, 35]	4.1	3.92	0%	40%	55%	Discard
Staff acknowledge/greet residents when they see them [7, 36, 42]	4.95	4.90	0%	75%	25%	Stay
Staff inquire about the residents family and visitors [unpublished pilot data]	4.40	4.28	3%	60%	38%	Discard
Staff visit with residents [36, 37]	4.75	4.75	3%	32.5%	65%	Discard
Staff make residents feel valued as a person [4, 7, 31, 32, 34–38]	4.90	4.85	3%	62.5%	35%	Stay
Staff listen to resident concerns [4, 7, 31, 34, 36–39]	4.95	4.95	0%	60%	40%	Stay
Staff speak to residents, not over them [4, 34, 39]	4.93	4.88	0%	42.5%	58%	Stay
Staff treat residents like family [unpublished pilot data]	3.85	4.03	23%	45%	28%	Discard
Staff do not make residents feel like a ‘burden’ to others [4, 8, 31, 36, 37]	4.98	4.93	0%	50%	48%	Stay
Staff address residents by the name they wish to be called [4, 32, 34, 36]	4.97	4.90	0%	87.5%	13%	Stay
Staff remember residents personal likes and dislikes [32]	4.77	4.79	3%	32.5%	60%	Discard
Cultural and religious preferences are accounted for [4, 7, 32, 34, 36, 37]	4.77	4.67	0%	42.5%	53%	Stay
Residents have control over how their personal space looks [4, 7, 8, 31]	4.72	4.56	5%	37.5%	55%	Discard (Making stay base on qualitative feedback)
Residents have control over how they wish to look [4, 8, 32, 36]	4.87	4.74	0%	60%	38%	Stay
Residents have control over how finances are handled (whether by self or elected proxy) [4, 35, 40]	4.49	4.28	5%	60%	30%	Discard
Birthdays are celebrated [unpublished pilot data]	4.58	4.51	0%	97.5%	3%	Discard
Residents have control over their end-of-life care plans [4, 35, 37]	4.88	4.75	3%	47.5%	50%	Stay
**Care and Assistance n = 15**						
Staff do not multitask when providing care [7]	3.98	4.08	15%	17.5%	65%	Discard
Assistance from staff is timely; residents are not made to wait [4, 7, 32, 34, 36]	4.55	4.54	18%	2.5%	78%	Discard
Staff is compassionate in providing care [7, 34, 35, 37]	5.00	4.93	0%	50%	50%	Stay
Food is presented appropriately [4, 7]	4.70	4.65	5%	65%	30%	Discard
The call bell is put within reach for residents [unpublished pilot data]	4.90	4.80	0%	87.5%	13%	Stay
Residents have a choice to have extra snacks [4, 38]	4.45	4.15	5%	65%	28%	Discard
Residents have a choice of what, where, and how to eat [4, 7, 38, 39]	4.41	4.41	18%	15%	68%	Discard
Residents have a choice of wash, shower, or bath [4, 39]	4.60	4.44	15%	40%	43%	Discard
Residents have control over their daily schedule (when to sleep, eat, bath, use the washroom) [4, 7, 35, 36, 38, 39]	4.63	4.69	15%	15%	70%	Discard (Making stay base on qualitative feedback)
Freedom exits to complain without fear of repercussions [7, 32, 34, 36]	4.98	4.85	3%	55%	40%	Stay
Residents have control over managing their own pain relief options [4, 32]	4.75	4.76	8%	37.5%	53%	Discard
More than one bath is allowed per week [4]	4.40	4.25	13%	25%	63%	Discard
The facility is kept clean and pleasant [4, 32]	4.88	4.78	0%	87.5%	13%	Stay
Equipment is available to maximize independence [4, 20]	4.85	4.78	3%	57.5%	40%	Stay
Specialized assistance is provided for those with disabilities (e.g. hearing loss) [4, 36]	4.77	4.79	3%	35%	63%	Discard
**Privacy n = 17**						
Assistance with hygiene and personal matters is appropriate and sensitive [4, 32, 34, 41]	4.93	4.88	0%	67.5%	33%	Stay
Permission is sought before physical contact [4, 7]	4.78	4.72	3%	57.5%	45%	Stay
Staff announce themselves before entering a residents room (e.g. knock) [4]	4.82	4.70	3%	80%	18%	Stay
Staff close curtains and doors [4, 32]	4.77	4.75	0%	90%	13%	Stay
Staff respect personal possessions [4, 39]	4.93	4.87	0%	75%	20%	Stay
Staff do not talk about residents in front of other residents (e.g. about their illness) [4, 32, 42]	4.98	4.85	0%	57.5%	45%	Stay
Efforts are made to respect modesty [4, 41]	4.85	4.75	0%	77.5%	20%	Stay
Residents can trust staff [32, 39]	4.98	4.90	3%	55%	38%	Stay
Residents have a choice of who assists with dressing, bathing, toileting (i.e. gender) [4, 41]	4.39	4.43	23%	25%	53%	Discard
Freedom exists to care for own personal hygiene if able [4]	4.93	4.87	0%	75%	25%	Stay
Precautions are taken to protect personal information [4, 34, 35]	4.95	4.75	3%	87.5%	13%	Stay
Freedom exits to use the washroom rather diapers [7]	4.88	4.87	3%	40%	50%	Stay
Private space is available to discuss sensitive matters [4]	4.88	4.83	3%	85%	13%	Stay
‘Do not disturb’ signs (or equivalent) are provided and respected [4]	4.50	4.55	8%	82.5%	10%	Discard
A system in place for wandering residents [unpublished pilot data]	4.80	4.78	5%	62.5%	35%	Stay
Efforts are made to make residents feel safe [7, 36]	4.98	4.85	0%	65%	35%	Stay
Single room accommodation is available [7, 36]	4.85	4.85	5%	60%	35%	Stay
**Social Interaction n = 14**						
Residents have a choice of whether or not to attend activities [4, 7, 36]	4.88	4.65	0%	92.5%	5%	Stay
Residents are able to make suggestions for facility activities [4]	4.73	4.54	0%	92.5%	8%	Stay
Residents are in control of when friends and relatives visit [4]	4.60	4.63	8%	75%	15%	Discard
There are numerous activities to select from (good rotation of activities) [38]	4.60	4.73	0%	65%	35%	Stay
There is space to visit with family and visitors [4]	4.90	4.82	0%	75%	25%	Stay
Transportation for community events is available [4]	4.55	4.43	5%	55%	43%	Discard
Group outings are available [4, 8, 38]	4.41	4.35	3%	70%	28%	Discard
Phone, computer, and internet access is provided [4]	4.21	4.15	3%	50%	43%	Discard
Programs are provided where community members come in to visit residents (e.g. children, volunteers) [unpublished pilot data]	4.55	4.63	0%	75%	23%	Discard
Visitors are warmly welcomed by staff [4, 7, 42]	4.64	4.65	0%	80%	18%	Discard
Resident to resident relationships are fostered [8, 36, 38]	4.77	4.65	0%	62.5%	33%	Stay
Staff do not speak amongst themselves in their native language in front of residents [unpublished pilot data]	4.71	4.65	3%	55%	40%	Stay
Programs are in place to overcome language barriers [32, 41]	4.44	4.45	5%	27.5%	65%	Discard
There are places for prayer, meditation, & spiritual counsel [4, 36]	4.74	4.59	0%	57%	35%	Stay

In Round 2 participants re-scored items using the same achievability scale and were instructed to pick the markers they would include in their top 15. Of the 38 markers, 10 were identified by >55% of respondents as being important to include in a final list of markers (Table 2).

**Table 2:** Scores for Round Two.

		Achievability, % No not at all (1)	Achievability, % "Yes, easily" (2)	Achievability, % Not easily achievable (3)	Percent who had it in top 15	Kept for Round 3 (Y/N)
**Being Known**	Staff acknowledge/greet residents when they see them	0	83	17	45	N
	Staff make residents feel valued as a person	3	61	33	60	Y
	Staff listen to resident concerns	0	75	25	65	Y
	Staff speak to residents, not over them	0	36	61	60	Y
	Staff do not make residents feel like a ‘burden’ to others	0	61	36	45	N
	Staff address residents by the name they wish to be called	0	94	6	55	Y
	Cultural and religious preferences are accounted for	0	56	44	15	N
	Residents have control over how their personal space looks (e.g. personal photos and possessions)	0	47	53	25	N
	Residents have control over how they wish to look	0	72	28	30	N
	Residents have control over their end-of-life care plans	0	44	56	30	N
**Care & Assistance**	Staff is compassionate in providing care	3	58	39	55	Y
	The call bell is put within reach for residents	0	94	6	20	N
	Residents have control over their daily schedule (when to sleep, eat, bath, use the washroom)	11	8	81	40	N
	Freedom exits to complain without fear of repercussions	0	61	39	40	N
	The facility is kept clean and pleasant	0	94	6	25	N
	Equipment is available to maximize independence	0	64	36	5	N
	Assistance with hygiene and personal matters is appropriate and sensitive	0	75	25	75	Y
**Privacy**	Permission is sought before physical contact	0	69	31	35	N
	Staff announce themselves before entering a residents room (e.g. knock)	0	89	11	25	N
	Staff close curtains and doors	0	94	6	25	N
	Staff respect personal possessions	0	8	92	35	N
	Staff do not talk about residents in front of other residents (e.g. about their illness)	0	58	42	50	Y
	Efforts are made to respect modesty	0	89	11	60	Y
	Residents can trust staff	0	53	42	55	Y
	Freedom exists to care for own personal hygiene if able	0	83	17	35	N
	Precautions are taken to protect personal information	0	94	6	20	N
	Freedom exits to use the washroom rather ‘diapers’ (adult briefs)	3	28	69	55	Y
	Private space is available to discuss sensitive matters	0	94	6	30	N
	A system in place for wandering residents	0	75	25	30	N
	Efforts are made to make residents feel safe	0	78	22	65	Y
	Single room accommodation is available	0	64	36	20	N
**Social Interaction**	Residents have a choice of whether or not to attend activities	0	100	0	30	N
	Residents are able to make suggestions for facility activities	0	89	11	30	N
	There are numerous activities to select from (good rotation of activities)	0	72	28	30	N
	There is space to visit with family and visitors	0	83	17	35	N
	Resident to resident relationships are fostered	0	75	25	20	N
	Staff do not speak amongst themselves in their native language in front of residents	3	58	39	40	N

In the final Delphi round, participants strongly and unanimously endorsed the 10 markers. However, qualitative comments from 72% of participants (26/36) indicated that 2 additional markers related to resident choice (e.g. residents are able to make choices in their everyday life) and privacy (e.g., residents personal space and need for privacy are respected) needed to be part of the final list. The 12 markers were vetted by the advisory panel, and based on their recommendation that several of the markers could be captured under a broader marker (e.g. Staff listen to resident concerns was subsumed under Staff make residents feel valued as a person) a final list of 10 dignity-conserving care markers was established and endorsed by the advisory group (Table 3).

**Table 3:** List of Final Dignity-Conserving Care Markers.

Staff make residents feel valued as a person	Staff are compassionate in providing care	Residents can trust staff
Staff do not make residents feel like a ‘burden’ to others	Assistance with hygiene and personal matters is appropriate and sensitive	Staff do not talk about residents in front of other residents
Residents are able to make choices in their everyday life	Freedom exists to complain without fear of repercussions	Residents personal space and need for privacy are respected
		Efforts are made to make residents feel safe

## Discussion

Using a modified three-round Delphi procedure and the expertise of our advisory group, we were able to develop a comprehensive set of markers that capture the range and diversity of important dignity-conserving care strategies for use in NHs. The final 10 markers were judged as having high face validity by experts in the field and have explicit implications for enhancing the provision of daily dignified care to NH residents. These markers make an important first step towards identifying key areas of inquiry into the delivery of dignity-conserving care and can bolster the traditional quality indicators used in the NH setting to bridge an important gap in addressing the psychosocial and the less easily quantified needs of NH residents.

It is interesting to reflect on those markers that were scored as less achievable or of lower importance by the Delphi panellists. The majority of these fall within the social interaction domain, and indeed none of these items were rated as being important to include in the top 15 items. In some ways this is not surprising, given that social interaction is more about the lived experience of another; a perspective that has been noted as difficult to imagine [30]. The items that were identified as less achievable or of lower importance tended to be instances that are more about reflecting on and imagining the reality of another and not about what a healthcare provider can do for the resident. An important next step of this research will be to vet these markers with residents; a phase that will be reported on in a subsequent publication.

The final list of 10 dignity-conserving care markers identified in this study resonate with previous work conducted examining the support or preservation of dignity for older adults. NH residents describe factors that either preserve or undermine their personal dignity as including waiting for help, being undervalued, having choice, being treated with respect, being listened to, attending to the small details in care, and having a sense of control [8,9,31]. In their thematic analysis of empirical and theoretical literature, Gallager and colleagues identified four common themes regarding dignity could be captured under: environment of care; staff attitudes and behaviours; culture of care; and specific care activities [32]. These themes are echoed in the work conducted by the Picker Institute which identified choice, control, staff attitudes, and facilities as themes that cut across their indicators of dignity [4]. Both frameworks have significant overlap and the final list of 10 markers generated in this study can be captured within each. For example, *residents personal space and need for privacy are respected* fits within the theme of the environment of care [32] and control [4].

The attitudes and behaviours of staff are highly influential on whether resident dignity is bolstered or fractured. Good professional care that preserves dignity treats residents with attention and respect, listens to them and takes them seriously, gives them time, and values them [8]. In this regard, all the dignity-conserving care markers have some direct bearing on staff attitudes and their behaviours. For example, *staff do not make residents feel like a ‘burden’ to others*, requires staff to engage with residents and find ways to assist residents feel like productive members of the NH community. Having dignity-conserving care markers that require staff to become consciously aware of their influence on resident dignity is a vital step to ensure personal dignity is supported in this context.

While the concept of dignified care is highly complex, in essence delivering care that conserves dignity is about providing individualised care, which requires NH staff to *value the resident as a person* and to get to know who they are, their preferences, needs, and wishes [9]. The measurement of dignity-conserving care needs to reflect these intricacies and complexities of care in order to capture not only what is done in care settings but how it is done [4]. To this end, the measuring of dignity-conserving care will require a multi-pronged strategy that relies on observation, care provider, and care recipient reports. Further work is required to provide operational definitions of each care marker and examples of best practices in each domain and to ensure they hold value for NH residents themselves.

## Limitations

While this study has many strengths, including the selection and representation of diverse NH settings and care providers on the expert panel, [33] and the alignment of the findings with previously published research on NH resident dignity, some limitations should be acknowledged. First, while taking steps to ensure a robust search of the literature, our search strategy may have failed to include all the relevant literature on dignity and NH residents. Second, a limitation of the Delphi process is the general lack of agreement on the size of the expert panel, the criteria to define consensus and the potential to prematurely stop the rounds [24]. Finally, while we include literature from the perspective of NH residents and what they deemed important to their sense of dignity, no residents were part of the Delphi panel. As evidence suggests their perspective may differ as to what is valued in terms of care processes, it is therefore imperative that the next step is to validate this set of dignity-conserving care markers with NH residents.

## Conclusion

Developing markers of dignity-conserving care specific to the NH setting is an important step in improving care of older adults within this milieu. These markers hold value for educational and benchmarking purposes and ultimately, will assist in operationalizing strategies for improving dignity-conserving care in NHs.
